# Effect of tryptophan residues on gold mineralization by a gold reducing peptide†

**DOI:** 10.1039/d0ra07098j

**Published:** 2020-11-06

**Authors:** Makoto Ozaki, Shuhei Yoshida, Maho Oura, Takaaki Tsuruoka, Kenji Usui

**Affiliations:** Faculty of Frontiers of Innovative Research in Science and Technology (FIRST), Konan University Kobe Japan kusui@konan-u.ac.jp

## Abstract

AuBP1, obtained by phage display selection, was previously shown to produce gold nanoparticles without reducing agents. The tryptophan (Trp) residue located at the N-terminus of this peptide contributes to the reduction of Au^3+^ to Au^0^ and is involved in the nucleation and crystal growth of gold nanoparticles. However, clear guidelines for relationships between the number of Trp residues in the peptide and its gold reducing ability have not been established. We focused on gold mineralization and attempted to elucidate aspects of the underlying mechanism. We performed a detailed evaluation of the effects of modifying the N-terminus of the core sequence on gold mineralization without reducing agents. Besides, advantages of utilizing peptides in manufacturing gold nanoparticles are shown. UV-Vis measurements, TEM observations, and kinetic analyses were used to show that increasing the number of Trp residues in the peptide increases the reducing ability, causing predominance of the nucleation reaction and the production of small gold nanoparticles. In addition, these peptides also had the ability as a dispersant to protect the surface of gold nanoparticles. Furthermore, the catalytic activity of mineralized gold nanoparticles with peptides was higher than that of a commercial gold nanoparticle. This study should help to elucidate the relationship between peptide sequence and mineralization ability for use in materials chemistry.

## Abstract 2

Increasing the number of tryptophan (Trp) in peptides led to higher gold reducing ability and the peptides could disperse the generated gold-nanoparticles.

## Graphical abstract

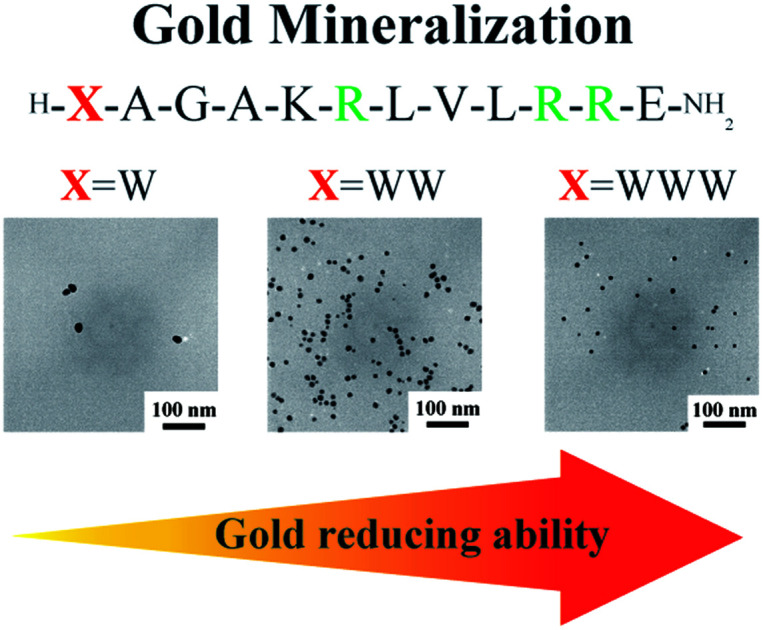

## Introduction

Gold nanoparticles have unique optical properties, and have been used in various applications such as nanotechnology,^1–3^ electronics,^4–6^ and biotechnology.^7–9^ However, the conventional synthesis methods, such as hydrothermal synthesis and chemical reduction, require environmentally hazardous substances, such as reducing agents, and consume a considerable amount of energy to achieve high temperature and high pressure. Mimicking biological systems is one of the most powerful approaches to overcoming these problems. For example, biomineralization (precipitation of inorganic compounds) systems can provide various inorganic precipitates following the addition of organic compounds such as proteins and peptides.^10–25^ The reaction conditions for the production of inorganic compounds in biomineralization systems are much milder than those used in conventional methods. Therefore, such inorganic compounds formed by mineralization have a low environmental load and reduced energy consumption. Recent research into the use of organic compounds such as proteins and peptides to precipitate inorganic compounds has shown that the resulting precipitates can be controlled with high reproducibility and accuracy. Among the organic compounds tested, peptides have various advantages, and are promising as materials for inorganic precipitation. (1) Small peptides derived from the sequences of proteins isolated for mineralization or artificial sequences can precipitate many types of inorganic compounds.^10–25^ (2) Peptides can be conjugated with artificial amino acids and functional molecules.^19,26–28^ (3) Peptides are easier to design and handle than proteins. However, clear guidelines for the relationships between peptide sequences and inorganic precipitation ability have not been established. Besides, other advantages of utilizing peptides in manufacturing inorganic nanoparticles have not been unveiled.

In this study, we focused on gold mineralization, and attempted to elucidate of the mechanism underlying gold ion (Au^3+^) reduction. Tan *et al.*, have shown that phenylalanine, tyrosine and tryptophan residues with aromatic groups are the most promising to reduce Au^3+^ to Au^0^.^29^ For example, a gold reducing peptide with a Trp residue was previously shown to produce gold nanoparticles without reducing agents.^30^ It has been reported that Trp residue contributes to the reduction of Au^3+^ to Au^0^ and is involved in the nucleation and crystal growth of gold nanoparticles.^31,32^ Consequently, here, we substituted a continuous sequence of Trp for the single Trp residue at the N-terminus in the core sequence (AuBP1) and performed a detailed evaluation of the effect on gold mineralization without reducing agents. We additionally demonstrated that the peptides also had the ability as a dispersant to protect the surface of gold nanoparticles. Finally, we evaluated the catalytic activity of mineralized gold nanoparticles with peptides in the reduction reaction of 4-nitrophenol to 4-aminophenol.

## Results and discussion

**Fig. 1:**
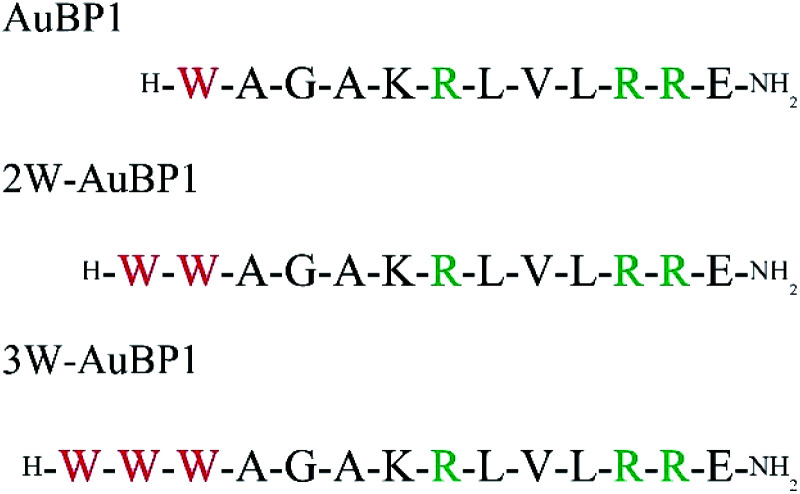
Sequences of gold reducing peptide used in this study.

First, we designed the peptides. The AuBP1 sequence (WAGAKRLVLRRE) obtained by phage display selection has a high affinity for Au surfaces and rivals the binding of alkanethiol on Au.^33^ In addition, this peptide was shown to produce gold nanoparticles without reducing agents. Arginine (Arg) residues located at positions 6, 10 and 11 of this peptide contribute to binding Au^3+^, and the Trp residue located at the N-terminus of this peptide contributes to the reduction of Au^3+^ to Au^0^.^31,32^ We also modified the core sequence by substituting a continuous sequence of Trp residues for the single Trp residue at the N-terminus (Fig. 1).

We first confirmed the secondary structures of three peptides (AuBP1, 2W-AuBP1, and 3W-AuBP1) by CD measurements. CD measurements showed that all the peptides formed similar random-coil-like structures (Fig. S1, ESI†). These results suggested that the potential influence of peptide secondary structural differences on gold mineralization can be excluded. Therefore, we can concentrate on the number of Trp residue in discussing about the effect of gold mineralization.

**Fig. 2:**
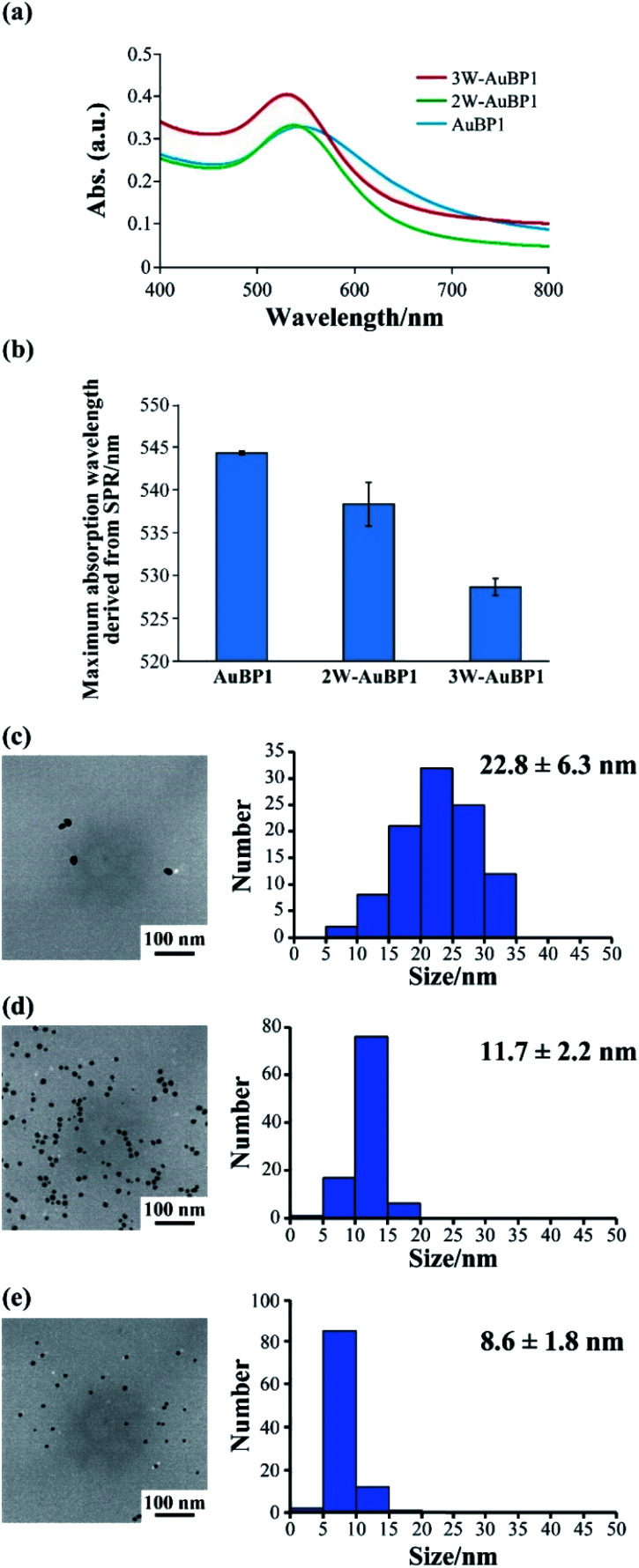
(a) UV-Vis spectra of the sample after gold reduction using peptides. (b) Maximum absorption wavelength of the sample after gold reduction for 24 h using peptides. TEM images (left) and particle size histograms (right) of the sample after gold reduction for 24 h using (c) AuBP1, (d) 2W-AuBP1, and (e) 3W-AuBP1.

Next, we estimated the abilities of these peptides to reduce Au^3+^ by measuring the absorption derived from surface plasmon resonance (SPR) using UV-Vis spectroscopy. At a peptide concentration of 100 μM, absorption derived from SPR at approximately 520–540 nm was confirmed for all peptides (Fig. 2a). Thus, the absorbance at each time was plotted, and the gold reducing ability was compared for each peptide. The results showed that as the number of Trp residues increased, the absorption derived from SPR was confirmed closer to the beginning of the reaction (Fig. S2, ESI†), suggesting that increasing the number of Trp residues increased the gold reduction efficiency. Furthermore, the maximum absorption wavelength of the samples after incubation for 24 h was compared for each peptide. As the number of Trp residues increased, the absorption peak derived from SPR was confirmed to undergo a blueshift (Fig. 2b). These results imply that the size of the gold nanoparticles formed by peptides with large number of Trp residues was small.

The differences in size and shape of the formed gold nanoparticles were analyzed by TEM. The TEM observations showed that using peptides with a large number of Trp residues produced gold nanoparticles of small size (Fig. 2c and S3a, ESI:† AuBP1 produced gold nanoparticles with diameters of 22.8 ± 6.3 nm; Fig. 2d and S3b, ESI:† 2W-AuBP1 produced gold nanoparticles with diameters of 11.7 ± 2.2 nm; Fig. 2e and S3c, ESI:† 3W-AuBP1 produced gold nanoparticles with diameters of 8.6 ± 1.8 nm). The size distribution of the formed gold nanoparticles became smaller as the number of Trp residues increased. These results suggested that peptides with a larger number of Trp residues have higher reducing ability, causing the nucleation reaction to predominate and small gold nanoparticles to be produced.

**Fig. 3:**
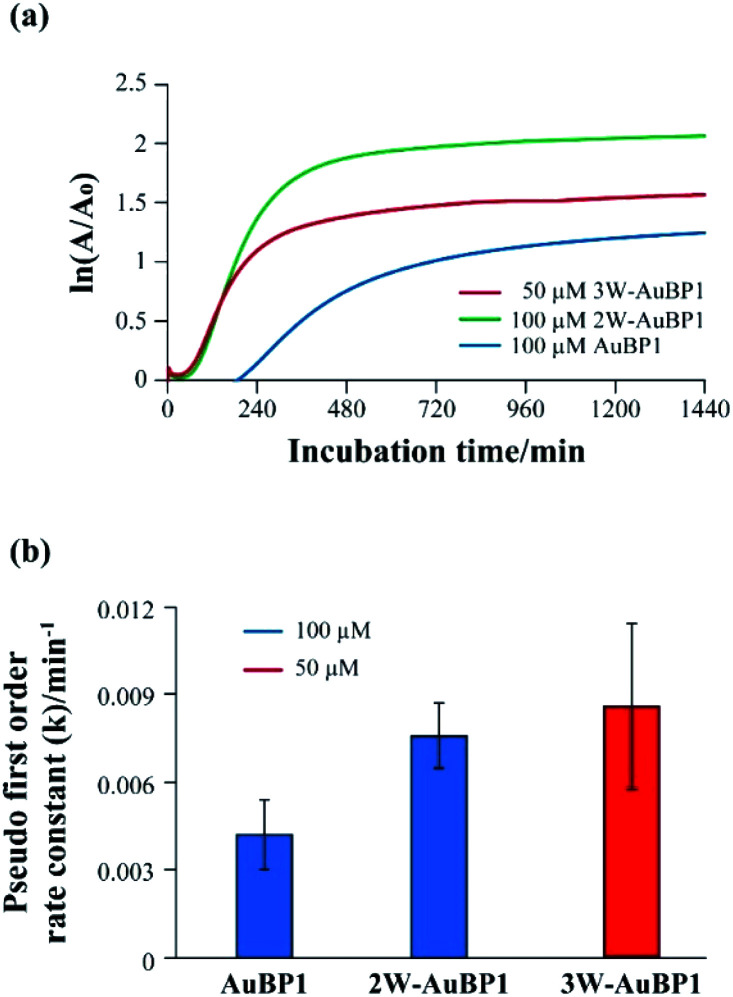
(a) Pseudo-first-order kinetic analysis of the reduction of Au^3+^ in the presence of peptides. (b) Calculated *k* values for the reduction of Au^3+^ in the presence of peptides.

Subsequently, the gold reducing ability of each peptide was clarified by calculating the reaction rate constants (pseudo-first-order rate constants) of the gold reduction reaction. Fig. S2† shows the growth of the SPR monitored at 540 nm over time for each peptide. The natural log of the absorbance at 540 nm, divided by the initial absorbance at this wavelength, was plotted as a function of time (Fig. 3a). The data were subsequently fit with a linear best fit line whose slope represents the pseudo-first-order rate constant. The *k* values of the peptides were (4.2 ± 1.2) × 10^3^ min^−1^ (AuBP1) and (7.6 ± 1.1) × 10^3^ min^−1^ (2W-AuBP1). The *k* value of 2W-AuBP1 was higher than that of AuBP1 (Fig. 3b). In the sample with 3W-AuBP1, gold nanoparticles were formed immediately after the addition of HAuCl_4_. Therefore, the absorbance rapidly increased from the initial stage of the reaction, and it was difficult to accurately calculate the rate constant (Fig. S4, ESI†). Thus, the pseudo-first-order rate constant for 3W-AuBP1 was calculated at 50 μM (Fig. 3a) to be (8.6 ± 2.8) × 10^3^ min^−1^ (Fig. 3b). Despite the 2-fold lower concentration, the *k* value of the sample with 3W-AuBP1 was higher than that of 2W-AuBP1. These results showed that increasing the number of Trp residues increased the gold reduction rate of Au^3+^ to Au^0^.

**Fig. 4:**
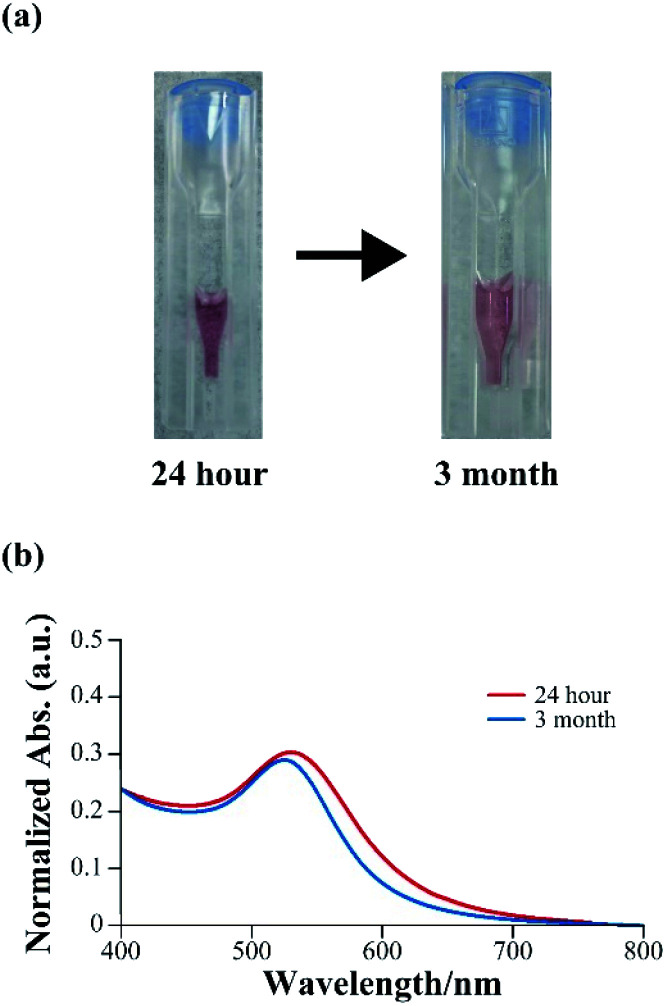
(a) Photograph of mineralized gold nanoparticles obtained with 100 μM 3W-AuBP1 after 24 h, and 3 months. (b) UV-Vis spectra of the sample after gold mineralization using 3W-AuBP1 after 24 h, and 3 months.

Synthesized gold nanoparticles generally require a dispersant to prevent aggregation. However, mineralized gold nanoparticles obtained using 3W-AuBP1, which had a high reducing ability, remained dispersed without aggregates even after 3 months (Fig. 4a). Thus, we confirmed using UV-Vis measurements that mineralized gold nanoparticles remained dispersed after 24 h and 3 months. The absorption peak derived from SPR in each sample changed only slightly (Fig. 4b). These results suggested that mineralized gold nanoparticles maintained a dispersed state. Furthermore, we measured the *ζ* potential in each sample. The gold nanoparticles incubated with peptide for 24 h had a negative *ζ* potential value (−28.4 mV), as expected. The sample incubated for 3 months showed almost the same negative potential value (−25.8 mV) as that of the 24 h-sample. These results suggested that these peptides not only had a gold reducing ability but exerted protective effect on the surface of gold nanoparticles.

Next, we gradually decreased the concentration of the peptide and examined the concentration thresholds at which Au^3+^ could be reduced for each peptide by UV-Vis measurements. The absorption derived from SPR did not show peptide concentrations less than 50 μM in AuBP1 (Fig. S5a, ESI†), less than 25 μM in 2W-AuBP1 (Fig. S5b, ESI†), or less than 10 μM in 3W-AuBP1 (Fig. S5c and d, ESI†) by UV-Vis measurements. Gold reduction occurred at increasingly low concentrations as the number of Trp residues increased. These results suggested that the reduction ability of these peptides towards Au^3+^ correlated with the number of Trp residues.

**Fig. 5:**
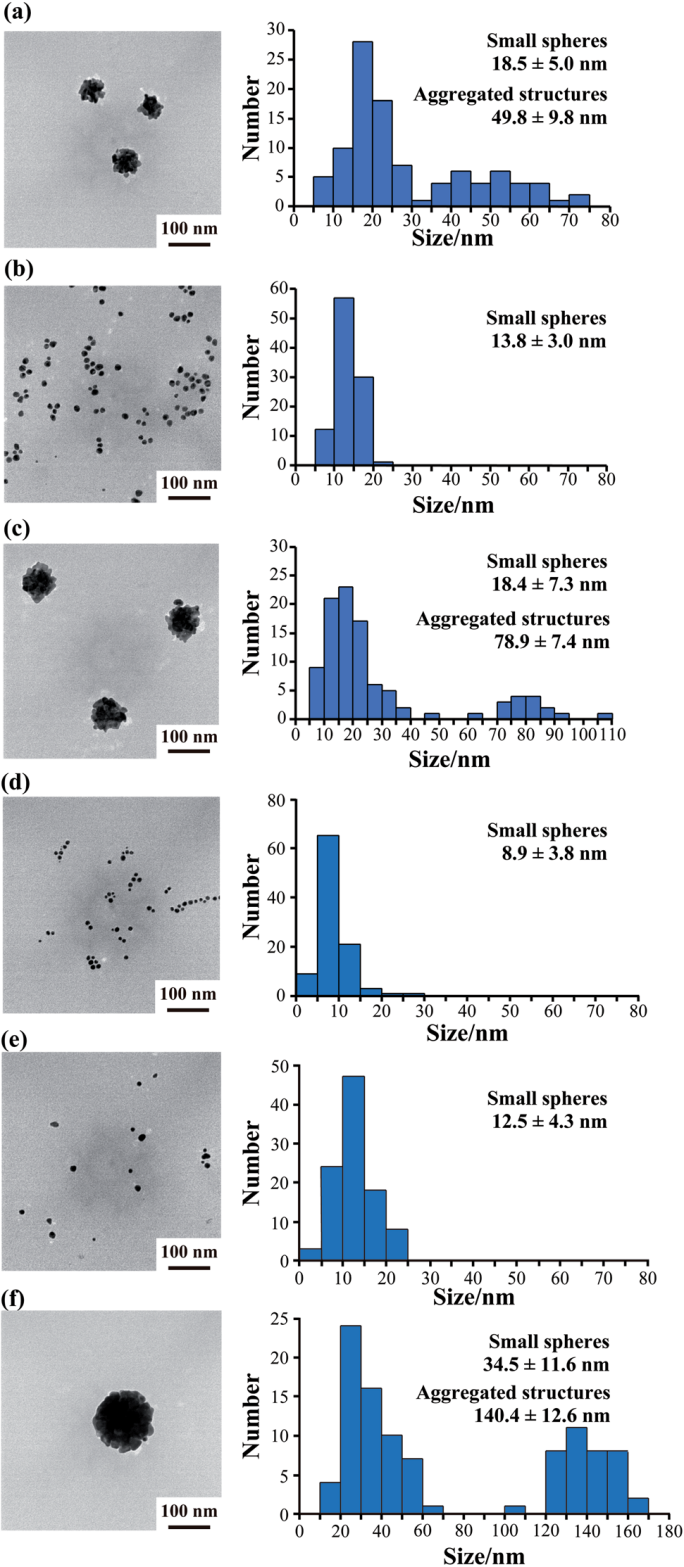
TEM images (left) and particle size histograms (right) of samples after gold reduction for 24 h using (a) 50 μM AuBP1, (b) 50 μM 2W-AuBP1, (c) 25 μM 2W-AuBP1, (d) 50 μM 3W-AuBP1, (e) 25 μM 3W-AuBP1, and (f) 10 μM 3W-AuBP1.

Furthermore, we observed the gold nanoparticles formed at various peptide concentrations by TEM. TEM observation at 50 μM AuBP1 showed 18.5 ± 5.0 nm small gold spheres and 49.8 ± 9.8 nm large aggregated structures (Fig. 5a and S6a, ESI†).

However, when 2W-AuBP1 and 3W-AuBP1 were used, TEM observation at 50 μM showed only small gold spheres (Fig. 5b and S6b, ESI:† 2W-AuBP1 showed gold spheres with diameters of 13.8 ± 3.0 nm; Fig. 5d and S6d, ESI:† 3W-AuBP1 showed gold spheres with diameters of 8.9 ± 3.8 nm). Comparing the particle diameter and size distribution of the gold spheres in the samples of 2W-AuBP1 and 3W-AuBP1 showed that the gold spheres obtained with 3W-AuBP1 were smaller than those obtained with 2W-AuBP1. The particle diameter and size distribution of gold nanoparticles decreased as the number of Trp residues increased, as at 100 μM peptide. TEM observations at 25 μM 2W-AuBP1 showed 18.4 ± 7.3 nm small gold spheres and 78.9 ± 7.4 nm large aggregated structures (Fig. 5c and Fig. S6c, ESI†). TEM observation at 25 μM 3W-AuBP1 showed only 12.7 ± 4.5 nm small gold spheres (Fig. 5e and S6e, ESI†). TEM observation at 10 μM 3W-AuBP1 showed only 32.9 ± 8.9 nm small gold spheres and 142.6 ± 12.1 nm aggregated structures (Fig. 5f and S6f, ESI†). These results showed that the peptides with a larger number of Trp residues have higher reducing ability, causing the nucleation reaction to predominate and gold nanoparticles with a small size and uniform size distribution to be produced.

Finally, we measured catalytic activity of mineralized gold nanoparticles using reduction reaction of 4-nitrophenol to 4-aminophenol. In general, the gold nanoparticles with size of 2 nm to 6 nm have high catalytic activity.^34,35^ Therefore, we used 5.9 ± 1.1 nm gold nanoparticles synthesized with trisodium citrate as a control sample (Fig. S7, ESI,† Sigma-Aldrich, 741949) and compared the catalytic activity of mineralized gold nanoparticles. 4-Nitrophenol has a maximum absorption at 400 nm under basic conditions, and the absorption peak is decreased by reduction of 4-nitrophenol to 4-aminophenol by NaBH_4_. Therefore, the reaction rate can be calculated from this decrease in absorption intensity at 400 nm using UV-Vis spectrophotometer. As shown in Fig. S8a (ESI†) the absorption intensity at 400 nm did not decrease as a function of time without gold nanoparticles. On the other hand, the absorption intensity at 400 nm of the mineralized gold nanoparticles and control sample decreased as a function time (Fig. S8b, ESI†). These results showed that gold nanoparticles catalyzed the reduction reaction of 4-nitrophenol to 4-aminophenol. The rate constants in the reduction reaction of 4-nitrophenol were calculated from the slope of the linear region of the obtained plot from the experiment. The activation energy (*E*_a_) values were obtained by creating an Arrhenius plot from the rate constants obtained by conducting a catalytic activity experiment while changing the reaction temperature from 20 °C to 40 °C (Fig. S9a–c, ESI†). The *E*_a_ value of the control sample was 70.9 kJ mol^−1^ (Table 1). The *E*_a_ values of the mineralized gold nanoparticles were 60.1 kJ mol^−1^ (AuBP1), 56.0 kJ mol^−1^ (2W-AuBP1), and 54.5 kJ mol^−1^ (3W-AuBP1) (Table 1). The mineralized gold nanoparticles had higher catalytic activity than that of the commercial gold nanoparticle. In addition, increasing the number of Trp residues increased catalytic activity. As the number of Trp residues increases, the specific surface area also increases due to the smaller size of the mineralized gold nanoparticles. These results and the dispersion results in Fig. 4 suggested that the peptides on the nanoparticles interrupted the aggregation of the mineralized gold nanoparticles created new active sites and simultaneously adsorbed hydrogen and 4-nitrophenol.

**Table 1:** Activation energy (*E*_a_) values of mineralized gold nanoparticles and the commercial gold nanoparticle

	5 nm AuNPs	AuBP1	2W-AuBP1	3W-AuBP1
Activation energy (*E*_a_)	70.9 kJ	60.1 kJ	56.0 kJ	54.5 kJ

Furthermore, we observed the gold nanoparticle morphology using TEM after the reduction reaction. Although TEM images showed that some aggregated nanoparticles were observed, the dispersed state seemed to be maintained to some extent (Fig. S10, ESI†). These results implied that these peptides exerted some protective effect on the surface of gold nanoparticles even during the reduction reaction. Peptides with higher reduction and dispersing effect would produce gold nanoparticles with higher catalytic activity.^36^

## Conclusions

We showed that the gold reducing activities of three derivatives of AuBP1 correlate with the number of Trp residues located at the N-terminus. UV-Vis measurements, TEM observations, and kinetic analyses were used to show that increasing the number of Trp residues in the peptide increases the reducing ability, causing predominance of the nucleation reaction and the production of small gold nanoparticles. In addition, these peptides exerted protective effect toward the surface of mineralized nanoparticles. Furthermore, mineralized gold nanoparticles had high catalytic activity than that of the commercial gold nanoparticle. Consequently, elucidating the relationship between the peptide sequence and the ability to reduce Au^3+^ will be indispensable for controlling precipitate morphologies in the near future. This information should help to elucidate the relationship between peptide sequence and mineralization ability, including deposit morphology and reducing activity, for use in nanobiochemistry and materials chemistry research.

## Materials and methods

### General remarks

All chemicals and solvents were of reagent or HPLC grade and were used without further purification. HPLC was performed on a GL-7400 HPLC system (GL Sciences, Tokyo, Japan) using an Inertsil ODS-3 column (10 × 250 mm; GL Science) for preparative purification, with a linear acetonitrile/0.1% trifluoroacetic acid (TFA) gradient at a flow rate of 3.0 mL min^−1^. The peptides were analyzed using MALDI-TOF MS on an Autoflex III (Bruker Daltonics, Billerica, MA, USA) mass spectrometer with 3,5-dimethoxy-4-hydroxycinnamic acid as the matrix. Amino acid analysis was carried out using an Inertsil ODS-2 column (4.6 × 200 mm; GL Science) after samples were hydrolyzed in 6 M HCl at 110 °C for 24 h in a sealed tube and labeled with phenyl isothiocyanate.

### Peptide synthesis

The designed peptides were synthesized manually on Fmoc-NH-SAL-PEG resin (Watanabe Chemical Industries, Hiroshima, Japan) using Fmoc chemistry^37^ with Fmoc-AA-OH (5 eq., Watanabe Chemical Industries) according to the *O*-(7-azabenzotriazol-1-yl)-1,1,3,3-tetramethyluronium hexafluorophosphate (HBTU, Watanabe Chemical) method. Side-chain protection was as follows: *t*-butyl (*t*Bu) for Ser, *t*-butyloxycarbonyl (Boc) for Lys, 2,2,4,6,7-pentamethyldihydrobenzofuran-5-sulfonyl (Pbf) for Arg. The peptides were cleaved from the resins, and the side-chain protection was removed by incubating the peptides for 1 h in H_2_O/triisopropylsilane (Wako Pure Chemical Industries, Tokyo, Japan)/thioanisole/TFA (Watanabe Chemical Industries) (1 : 1 : 1 : 50, v/v). The peptides were then precipitated by the addition of cold diethyl ether, collected by centrifugation, purified by RP-HPLC, and characterized by amino acid analysis and MALDI-TOF MS: AuBP1, *m*/*z* 1453.9 ([M + H]^+^ calc. 1543.8); 2W-AuBP1, *m*/*z* 1640.9 ([M + H]^+^ calc. 1640.0); and 3W-AuBP1, *m*/*z* 1826.9 ([M + H]^+^ calc. 1826.2) (Fig. S11, ESI†). The purified peptides were dissolved in MilliQ water to approximately 1 mM, and their concentrations were determined by amino acid analysis. The peptides were stored at 4 °C.

### CD measurements

CD spectroscopy was performed at r.t. using 200 μL of 10 μM peptides. A J-820 spectropolarimeter (JASCO) with a thermoregulator and a quartz cell with a 0.2 cm path length was used. Molecular ellipticities represented mean residual values calculated by the number of peptide bonds (12 for AuBP1, 13 for 2W-AuBP1, 14 for 3W-AuBP1).

### Gold mineralization using peptides

In a UV cuvette cell (Merck, BR759200) with a 1 cm pathlength, 35 μL of 1.0 mM peptide solution was diluted in 280 μL of MilliQ water. To this mixture, 35 μL of 1.0 mM HAuCl_4_ was added, and the mixture was gently stirred. The sample solution (350 μL) was incubated for 24 h at r.t.

### UV-Vis measurements and kinetic analysis

The absorption peak derived from SPR of the sample after gold mineralization for 24 h was measured using UV-1800 spectrometer (Shimadzu, Kyoto, Japan). In kinetic analysis, the growth in the absorbance derived from SPR at 540 nm for each peptide was monitored over time for 24 h. The natural log of the absorbance at 540 nm was divided by the initial absorbance at this wavelength and plotted as a function of time. The data were subsequently fit with a linear best fit line whose slope of represents the pseudo-first-order rate constant.

### TEM measurements

After gold mineralization (5–100 μM peptide, HAuCl_4_ 100 μM), 20 μL samples were placed on TEM grids (Cu 200 mesh covered with a Nisshin EM collodion membrane; Nisshin, Tokyo, Japan). After 5 min, the solvent was absorbed with filter paper. MilliQ water (20 μL) was then placed on the TEM grid and immediately absorbed with filter paper. This process was repeated three times to remove salts from the sample. All samples were dried *in vacuo* before TEM measurements, which were conducted at an accelerating voltage of 115 kV (JEM-1400; JEOL, Tokyo, Japan).

### *ζ* potential measurements

Sample solution (750 μL for zeta potential) was transferred into a folded capillary cell DTS1070 (Malvern Instruments, Worcestershire, UK) for *ζ* potential measurements. *ζ* potential data were acquired on a Zetasizer ZEN3600 instrument (Sysmex, Kobe, Japan) equipped with a 633 nm laser.

### Catalytic activity

The sample solution (26 μL) was transferred into micro-multicell (208-92089, shimadzu, Kyoto, Japan) with a 1 cm pathlength and mixed with 52 μL of 7.5 mM NaBH_4_ in MilliQ water, then incubated for 10 min at r.t. This mixture was mixed with 32.5 μL of 400 μM 4-nitrophenol and 19.5 μL of 0.2 M Tris–HCl buffer (pH 8.5) (total volume 130 μL). The absorption intensity at 400 nm monitored as a function of time at 20 °C to 40 °C using UV-1800 spectrometer (Shimadzu, Kyoto, Japan).

## Conflicts of interest

The authors declare no conflicts of interest.

## Supplementary Material

RA-010-D0RA07098J-s001
