# Supplementary material for: Effect of tryptophan residues on gold mineralization by a gold reducing peptide
Source: RSC Adv. 2020 Nov 6;10(66):40461–6. doi: 10.1039/d0ra07098j (PMC9057565; doi:10.1039/d0ra07098j)
Supplement: RA-010-D0RA07098J-s001 [file RA-010-D0RA07098J-s001.pdf]

Electronic Supplementary Material (ESI) for RSC Advances.
This journal is © The Royal Society of Chemistry 2020

Supporting Information for

Effect of Tryptophan Residues on Gold Mineralization by a Gold Reducing Peptide

Makoto Ozaki, Shuhei Yoshida, Maho Oura, Takaaki Tsuruoka, and Kenji Usui*

[*] Dr. Kenji Usui, Mr. Makoto Ozaki, Mr. Shuhei Yoshida, Ms. Maho Oura, Dr. Takaaki Tsuruoka
Faculty of Frontiers of Innovative Research in Science and Technology (FIRST),
Konan University, 6500047, Kobe (Japan)
E-mail: kusui@konan-u.ac.jp (K. Usui)

CONTENTS:

Page S2: Fig. S1 for CD spectra of AuBP1, 2W-AuBP1, and 3W-AuBP1.
Page S3: Fig. S2 for growth of absorbance derived from SPR at 540 nm over time of each peptide
Page S4: Fig. S3 for TEM images of the sample after gold mineralization using 100 μM peptides
Page S5: Fig. S4 for kinetic analysis of reduction of Au^{3+} to Au^0 using 100 μM 3W-AuBP1
Page S6: Fig. S5 for UV-VIS data of the sample after gold mineralization using various concentration peptides
Page S7: Fig. S6 for TEM images of the sample after gold mineralization using peptides of various concentrations
Page S8: Fig. S7 for TEM image and particle histogram of the commercial gold nanoparticle
Page S9: Fig. S8 for catalytic activity data of reduction reaction of 4-nitrophenol with/without gold nanoparticles
Page S10: Fig. S9 for Arrhenius plots of the sample using mineralized gold nanoparticles
Page S11: Fig. S10 for TEM images of mineralized gold nanospheres using 100 μM peptides after reduction reaction of 4-nitrophenol
Page S12: Fig. S11 for HPLC and MS for purified AuBP1, 2W-AuBP1, and 3W-AuBP1

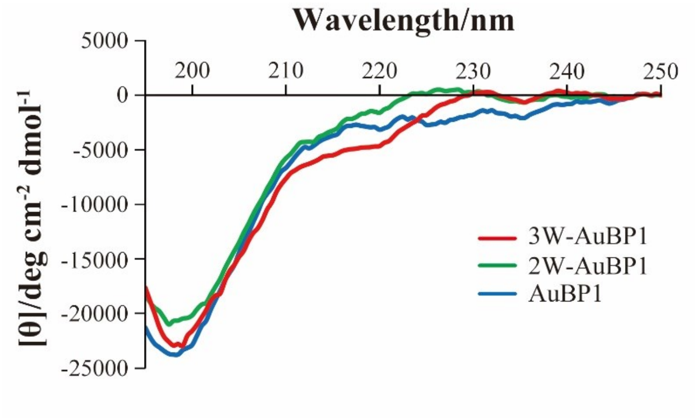

Fig. S1 CD spectra of 10 μM AuBP1, 2W-AuBP1, and 3W-AuBP1.

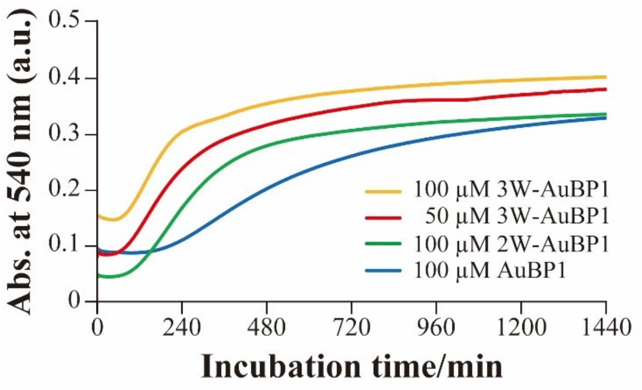

Fig. S2 Growth of absorbance derived from SPR at 540 nm over time of each peptide.

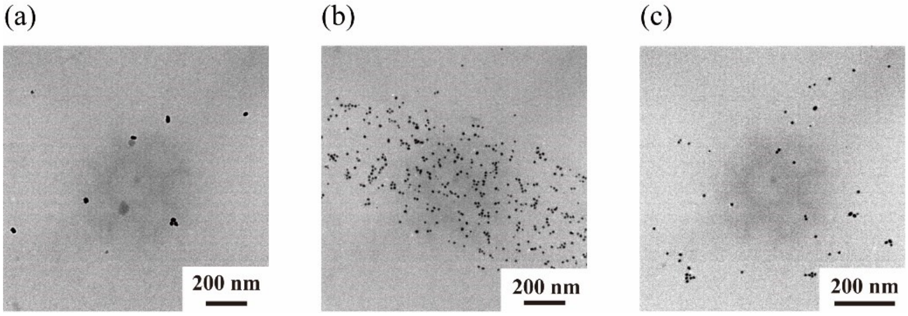

Fig. S3 TEM images of the sample after gold mineralization using (a) 100 µM AuBP1, (b) 100 µM 2W-AuBP1, and (c) 100 µM 3W-AuBP1.

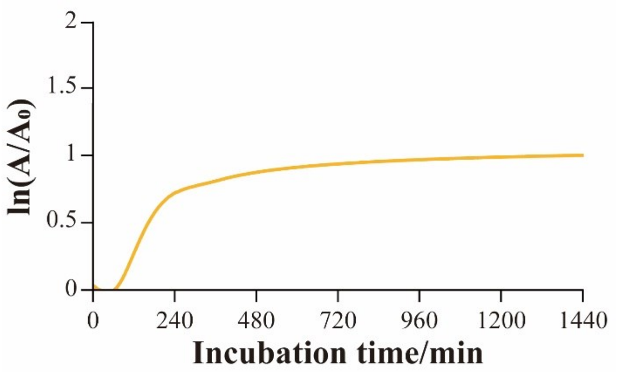

Fig. S4 Pseudo-first-order kinetic analysis of the reduction of Au^{3+} to Au^{0} using 100 μM 3W-AuBP1.

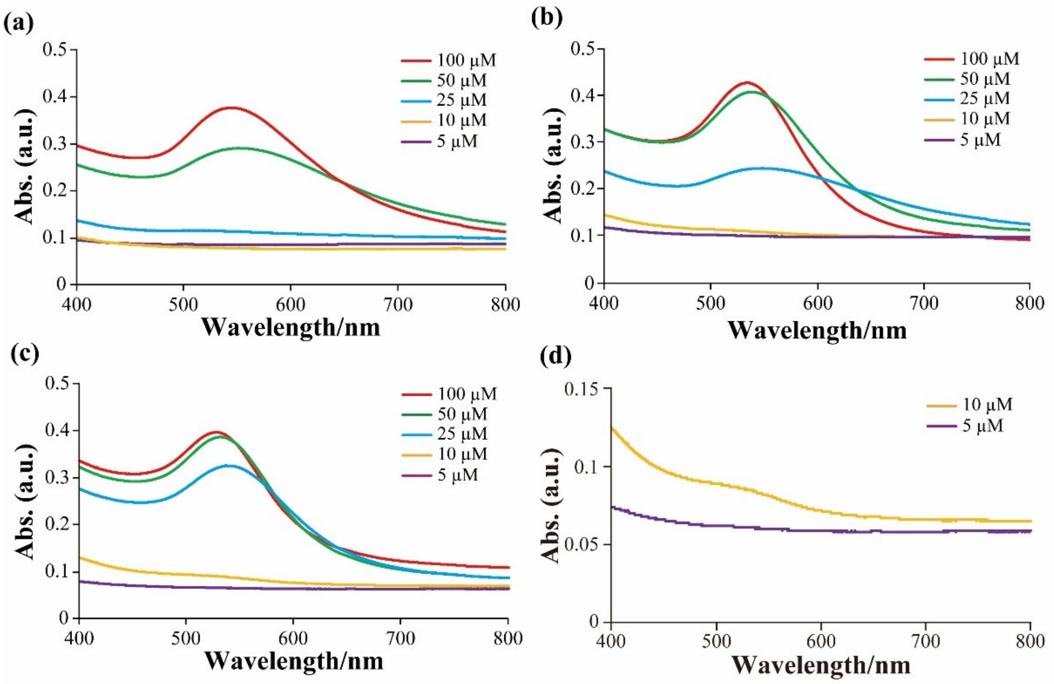

Fig. S5 UV-VIS spectra of the sample after gold mineralization using (a) AuBP1, (b) 2W-AuBP1, and (c), (d) 3W-AuBP1.

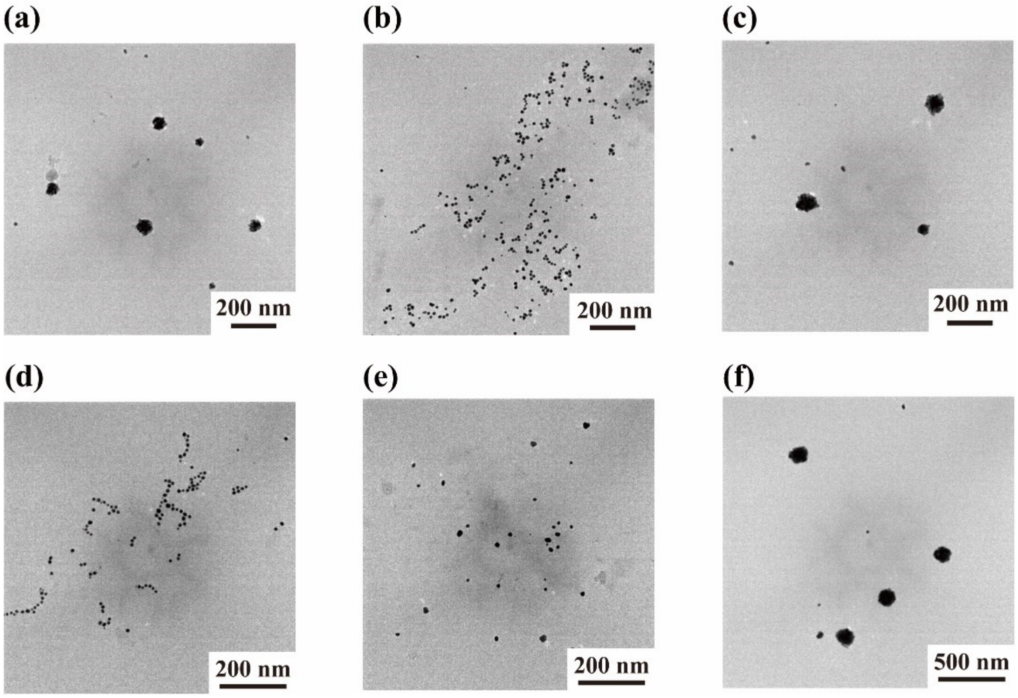

Fig. S6 TEM images of the sample after gold mineralization using (a) 50 μM AuBP1, (b) 50 μM 2W-AuBP1, (c) 25 μM 2W-AuBP1, (d) 50 μM 3W-AuBP1, (e) 25 μM 3W-AuBP1, and (e) 10 μM 3W-AuBP1.

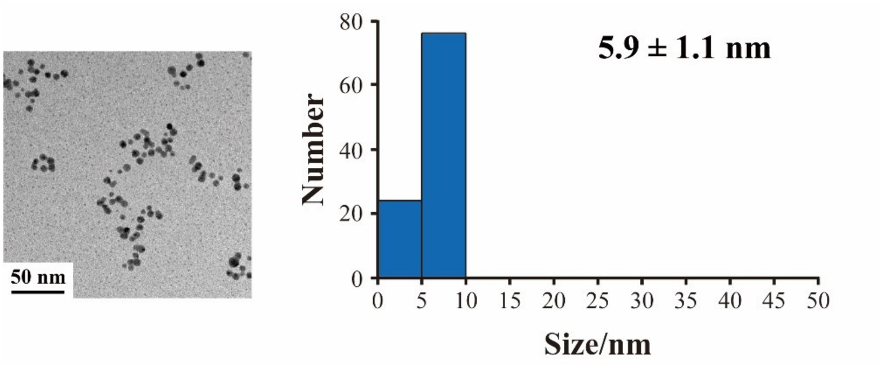

Fig. S7 TEM image and particle histogram of the commercial gold nanoparticle (Sigma-Aldrich, 741949).

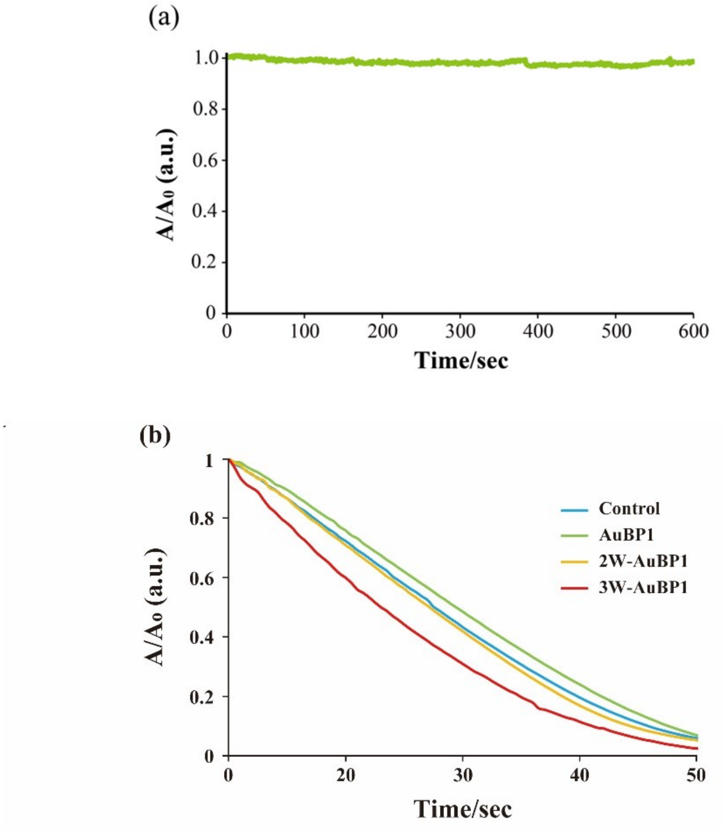

Fig. S8 (a) Time course of reduction reaction of 4-nitrophenol to 4-aminophenol without gold nanoparticles at 20°C. (b) Time course of reduction reaction of 4-nitrophenol to 4-aminophenol using gold nanoparticles at 30°C.

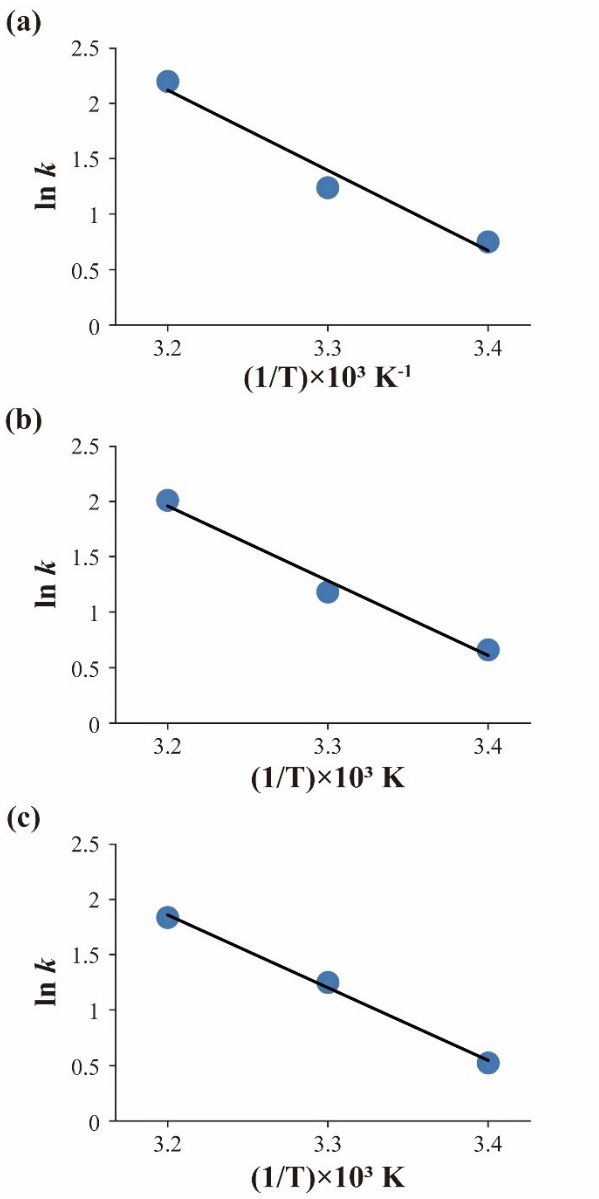

Fig. S9 Arrhenius plots of reduction reaction of 4-nitrophenol using (a) AuBP1, (b) 2W-AuBP1, and (c) 3W-AuBP1.

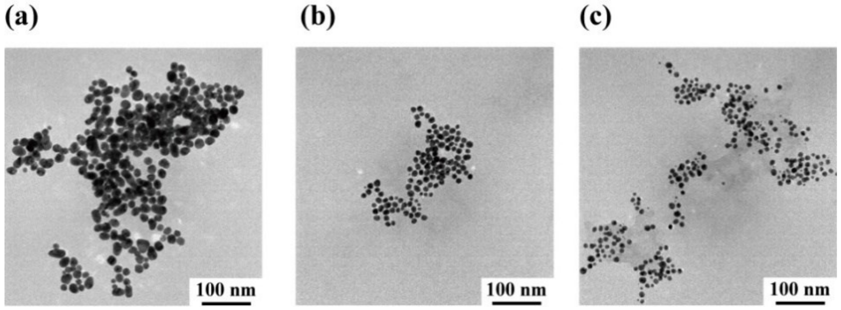

Fig. S10 TEM images of mineralized gold nanospheres with (a) 100 µM AuBP1, (b) 100 µM 2W-AuBP1, and (c) 100 µM 3W-AuBP1 after reduction reaction of 4-nitrophenol to 4-aminophenol is completed.

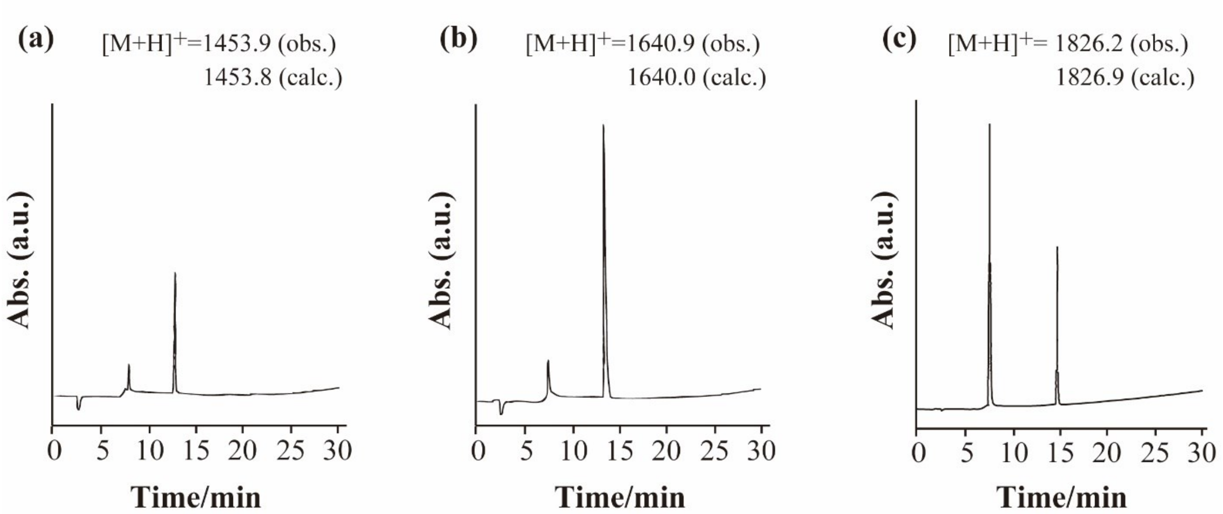

Fig. S11 HPLC for purified (a) AuBP1, (b) 2W-AuBP1, and (c) 3W-AuBP1 separated on an ODS column (150 × 4.6 mm, 5 mm) with MilliQ water (containing 0.1% TFA) using a gradient from 0% to 100% acetonitrile (containing 0.08% TFA) over 30 min, 1.0 mL/min; detection at 220 nm.
